# Switch from heterotrophy to autotrophy of apple cotyledons depends on NO signal

**DOI:** 10.1007/s00425-015-2361-x

**Published:** 2015-07-18

**Authors:** Urszula Krasuska, Karolina Dębska, Katarzyna Otulak, Renata Bogatek, Agnieszka Gniazdowska

**Affiliations:** Department of Plant Physiology, Warsaw University of Life Sciences–SGGW, Nowoursynowska Str. 159, 02-776 Warsaw, Poland; Department of Botany, Warsaw University of Life Sciences-SGGW, Nowoursynowska Str. 159, 02-776 Warsaw, Poland

**Keywords:** Chlorophyll *a* fluorescence, Photosynthesis, ROS, RuBisCO, Seed dormancy, Sugars

## Abstract

**NO accelerates transition of germinated embryos from heterotrophy to autotrophy by stimulation of chloroplasts maturation. NO-mediated autotrophy of apple seedlings correlates to increased content of RuBisCO small subunit and improvement of parameters of chlorophyll*a*fluorescence.**

Nitric oxide (NO) acts as signaling molecule involved in regulation of various physiological processes in plants, although its involvement in cotyledons greening is poorly recognized. To identify the importance of NO signal for plant growth and development we investigated the effects of short-term application of NO at various developmental stages of seedlings of apple (*Malus domestica* Borkh.) on cotyledons’ chlorophyll *a* to *b* ratio, chlorophyll *a* fluorescence, photosynthetic activity, carbohydrates and RuBisCO both subunits content. NO-dependent biochemical alterations were linked to cytological observation of developing plastids in cotyledons of apple plants. Abnormal plantlets developing from dormant apple embryos are characterized by anatomical malformations of cotyledons. Short-term pre-treatment with NO of isolated embryos or seedlings with developmental anomalies resulted in formation of plants with cotyledons of equal size and chlorophyll content; these responses were blocked by NO scavenger. NO independently of time point of application accelerated embryos transition from heterotrophy to autotrophy by stimulation of photosynthetic activity, improvement of parameters of chlorophyll *a* fluorescence (*F*_v_/*F*_m_, *F*_v_/*F*_0_) and increased content of RuBisCO small subunit. Further analysis showed that NO application modified glucose and hydrogen peroxide concentration in cotyledons. Beneficial effect of NO on development of seedlings without any abnormalities was manifested at ultrastructural level by decline in amount of proplastids and induction of formation and maturation of chloroplasts. Our data suggest that progress of autotrophy of young seedlings is governed by NO acting as stimulator of chloroplast-to-nucleus signaling.

## Introduction

Since the beginning of twenty first century, nitric oxide (NO) is one of the most frequently studied signaling molecules in plant cells. Due to specific features of gasotransmitters such as low molecular weight, high reactivity, ability for diffusion though biological membranes and lack of specific receptors it seems to be an important, key regulator of many physiological processes. Regulatory role of NO in plant ontogeny has been well documented starting from seed germination, while terminating at the stage of fruit ripening or leaves senescence (as review by Wang et al. 2013; Krasuska et al. 2015). NO has been also found to be involved in plant responses to various biotic and abiotic stresses (Misra et al. 2014; Yu et al. 2014), as a second messenger acting downstream of hormonal signaling cascades. Although, the number of papers referring to NO contribution in plant physiology is increasing rapidly, there are still relatively rare data concerning its impact on chloroplasts structure and function or photosynthetic metabolism in cotyledons (Procházková et al. 2013; Misra et al. 2014). An important function of NO in photosynthetic active organs, particularly leaves, is derived from its participation in ABA signaling in stomata guard cells (Ribeiro et al. 2008). There were several published papers that focused on protective action of exogenous donors of NO (mainly sodium nitroprusside—SNP) on function of photosynthetic apparatus under abiotic stress conditions (heat, salinity, drought or heavy metals) (Procházková et al. 2013; Misra et al. 2014).

Production of NO in plant cells occurs in different organelles: peroxisomes (Corpas et al. 2001), mitochondria (Gupta and Kaiser 2010), chloroplasts (Jasid et al. 2006; Tewari et al. 2013) or plasma membrane (Stöhr and Stremlau 2006). In general, the enzymatic NO biosynthesis in plants depends on nitrate/nitrite reduction or probably on l-arginine oxidation and has been reviewed in detail by Gupta et al. (2011) and Khan et al. (2013). Both pathways for NO generation have been demonstrated to function in photosynthetically active cells including guard cells (Misra et al. 2014) and particularly in chloroplasts (Jasid et al. 2006). Thus, there is no doubt on NO in vivo action in leaves or other organs containing plastids or proplastids, e.g., cotyledons. Scherer (2007) indicated high production of NO in cotyledons. Moreover, it was demonstrated that in cotyledons of soybean (*Glycine max* (L.) Merr.) NO content varied dependently on seedling age, with maximum at around 7th day of seedling development (Jasid et al. 2009). Various NO donors were confirmed to stimulate greening of etiolated seedlings (Zhang et al. 2006) or growth and greening of cotyledons (Gniazdowska et al. 2010a; Galatro et al. 2013). A close correlation between NO biosynthesis and chloroplast function was proved using Arabidopsis mutant *noa1*. Arabidopsis Nitric Oxide-Associated 1 (*NOA1*), was identified as *RIF1* (Flores-Perez et al. 2008). Nowadays, it is clear that NOA1 has a function distinct from NO synthesis (Crawford et al. 2006); however, supplementation with SNP improves the *rif1*-*1* growth phenotype (Flores-Perez et al. 2008). Nevertheless, the *rif1*-*1* allele of *noa1* was isolated due to defects in chloroplast biogenesis (Flores-Perez et al. 2008), which was rescued by sucrose and correlated with increased formation of fumarate (van Ree et al. 2011). Thus, it was proposed, that the reduced levels of photosynthates resulting from defective chloroplasts was the primary physiological defect of NOA1 loss of function (van Ree et al. 2011).

NO mode of action is thought to be associated with posttranslational modifications (PTMs) of proteins: *S*-nitrosylation or nitration. Numerous chloroplast proteins were identified as targets of NO action (Lindermayr et al. 2005), specifically those of the Calvin–Benson cycle and PSII proteins were pointed as targets of *S*-nitrosylation. Moreover, inactivation of RuBisCO by *S*-nitrosylation was also confirmed (Abat et al. 2008).

Apple (*Malus domestica* Borkh.) seeds are dormant, and do not germinate even in favorable conditions of temperature, moisture and light (Lewak 2011). Dormancy alleviation of apple seeds occurs after 90-day-long cold stratification and may be mimicked by short-term (3–6 h) pre-treatment of isolated embryos with various NO donors or cyanide (Gniazdowska et al. 2010b). Dormancy of apple embryos is expressed not only by inhibition of germination (restriction of elongation growth of radicle) but also as morphological abnormalities of cotyledons. In seedlings developing from dormant embryos, lower cotyledon (lying down on the wet base) is getting green and increasing in size, while the upper one remains white and is of constant (unchanged) dimension. It was demonstrated, in our previously published reports, that short-term pre-treatment of dormant apple embryos with reactive oxygen species (ROS) or NO, applied immediately after embryos isolation from seed coat overcomes formation of seedlings with anomalies, and results in growth of plantlets with two properly developed cotyledons (Gniazdowska et al. 2010b).

We suspect that greening of cotyledon after treatment with NO may be due to chloroplast differentiation and developmental reprogramming process leading to modification of chloroplastic electron transport chain and modulation of CO_2_ assimilation. By differing the moment of NO application at the beginning of embryo culture, or after formation of seedlings with malformation of cotyledons we created a useful model to explain an importance of NO in regulation of seedling development and formation and function of photosynthetic apparatus. The aim of our work was provided by studies using biochemical methods of determination of carbohydrate, ROS, chlorophyll level, accompanied by determination of photosynthetic activity and detection of RuBisCO subunit content with a background of cytological observation of ultrastructure of cotyledons’ cells.

## Materials and methods

### Plant material

**Fig. 1:**
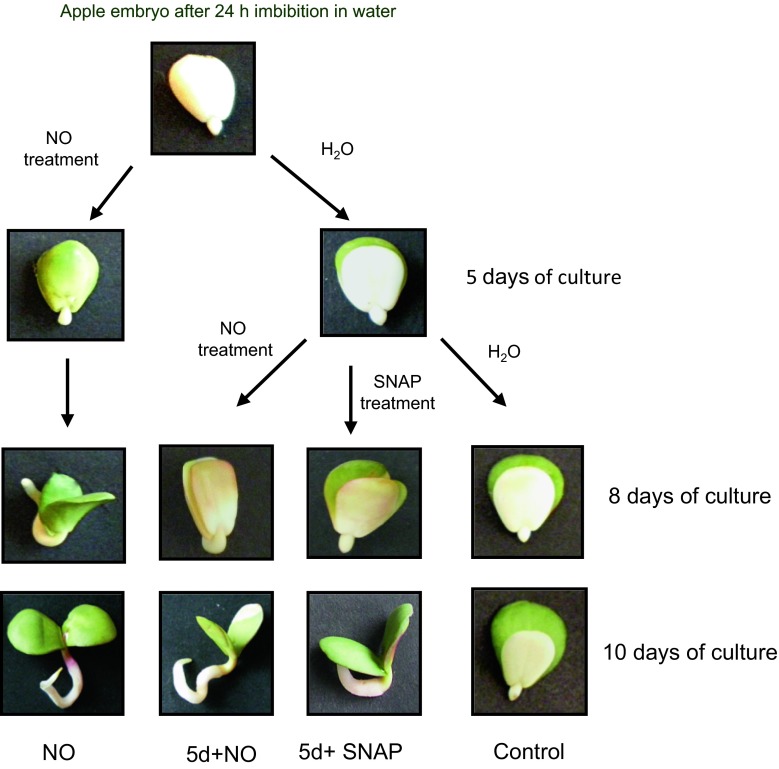
The scheme of apple embryos treatment with NO. Development of seedlings from control embryos (plants with morphological abnormalities) and embryos shortly treated after imbibition or after 5 days of culture with vapors of acidified nitrite (NO) or SNAP (plants with two cotyledons of equal size and greening)

As plant material apple (*Malus domestica* Borkh., cv. Antonówka, obtained from “Waldemar Andryka” commodity orchards) was used and embryos isolated from dormant seeds. Dormant seeds were stored in dark glass containers at 5 °C. Seed coat and endosperm were removed from seeds imbibed for 24 h in distilled water at room temperature. Embryos were shortly pre-treated with acidified nitrite, used as NO donor (Gniazdowska et al. 2010b). Acidified nitrite was prepared using 20 mM sodium nitrite (NaNO_2_) and 0.1 M HCl according to Yamasaki (2000) with some modifications. Embryos in lots of 60 were laid on filter paper moistened with 5 ml buffer 0.05 M Hepes–KOH pH 7.0 in the 500-ml glass chamber. A beaker containing 5 ml 20 mM NaNO_2_ was placed inside. Gaseous NO was produced by injecting 5 ml of 0.1 M HCl directly into the beaker with NaNO_2_. Embryos were exposed to vapors of acidified nitrite for 3 h in light. After NO treatment, embryos were washed twice in distilled water and placed (15 embryos per dish) on filter paper moistened with distilled water in glass Petri dishes (10 cm). As a control (C), isolated embryos were placed on filter paper wetted with distilled water. Part of the control embryos were collected after 5 days of culture and treated with NO (5d+NO) or *S*-nitroso-*N*-acetylpenicillamine (SNAP, 3 mM) solution for a short time (3 h) at light (SNAP treatment). The scheme of the experimental design is shown in Fig. 1. After treatment embryos were washed twice in distilled water and placed in 10-cm Petri dishes (15 embryos per dish) on filter paper wetted with distilled water or 0.3 mM 2-(4-carboxyphenyl)-4,4,5,5-tetramethylimidazoline-1-oxyl-3-oxide (cPTIO, NO scavenger) solution. Experiment with SNAP was provided only to determine impact of this NO donor on development of seedlings (growth and greening of cotyledons).

The culture of embryos was carried out in Sanyo growing chamber (Versalite Environmental Test Chamber MLR-35OH) at 25/20 °C with 12/12 h (light/dark) photoperiod, under light intensity 150 μmol PAR m^−2^ s^−1^.

For further analyses, seedlings [developed in water—control (C), developed from embryos shortly treated with NO (vapors of acidified nitrite) after imbibition (NO) or seedlings developed from the control seedlings shortly treated with NO (vapors of acidified nitrite) after 5 days of culture (5d + NO)] were collected after 8 and 10 days of growth (Fig. 1). After 5 days of culture only control (dormant embryos) and seedlings developing from embryos pre-treated with NO just after seed coat removal were used for determination.

### Hydrogen peroxide determination

Cotyledons separately isolated (upper and lower) from control and NO-treated seedlings after 5, 8 and 10 days of culture were used for H_2_O_2_ determination as described by Alexieva et al. (2001) with some modifications. Cotyledons (0.3 g) were homogenized with 0.1 % (w/v) trichloroacetic acid (TCA) in ice bath. The extract was centrifuged (MPW-350R centrifuge, MPW Med. Instruments, Warsaw, Poland) at 15,000*g* for 15 min at 4 °C. The supernatant was mixed with 0.1 % TCA, 10 mM potassium phosphate buffer pH 7.0, and freshly prepared 1 M potassium iodide (KI) in 10 mM potassium phosphate buffer pH 7.0. The H_2_O_2_ concentration was determined using Shimadzu UV 1700 spectrophotometer at 390 nm. Data were obtained in 4–5 independent experiments. The results were expressed as nmol mg^−1^ FW.

### Chlorophyll concentration measurement

Cotyledons (upper and lower) isolated separately from control and NO-treated seedlings after 5, 8 and 10 days of culture were collected and used for chlorophyll *a* and *b* measurement (Arnon 1949). Tissue (0.2 g) was homogenized in cooled mortar in 2 ml of 96 % ethanol with small amount of CaCO_3_ and immediately placed in the black tubes, then shortly mixed and centrifuged (15,000*g*, 10 min, MPW-350R centrifuge). Supernatant was used for chlorophyll determination at 645, 665, 694 nm by Hitashi U-2900 spectrophotometer. Chlorophyll *a* was calculated from the formula: 13.7*A*_665_ − 5.76*A*_694_, chlorophyll *b* from the formula: 25.8*A*_645_ − 7.6*A*_665_ (*A* means absorption rate at appropriate ƛ). Determination was done in 4–5 repetitions. The results were expressed as mg g^−1^ FW.

### Measurement of photosynthetic oxygen evolution

Clark-type oxygen electrode (Oxygraph 23107, Hansatech, Norfolk, UK) was used to estimate photosynthetic gas exchange. Before measurement seedlings were exposed for 15 min to 200 µmol PAR m^−2^ s^−1^. Then, seedlings were placed on distilled water in measurement chamber at temperature 25 °C, PAR—200 µmol m^−2^ s^−1^, and atmospheric CO_2_ concentration. After measurement was carried on light, seedlings were placed in the dark for 30 min and placed again in the chamber in the dark. Experiments were performed in 3–4 repetitions. Photosynthetic oxygen evolution was expressed as mmol O_2_ min^−1^ g^−1^FW.

### Chlorophyll *a* fluorescence measurement

Chlorophyll *a* fluorescence was measured at room temperature at ambient CO_2_ concentration using fluorometer (FluorCam 800MF, Photon System Instruments, Drasov, Czech Republic). Cotyledons collected separately from seedlings after 5, 8 and 10 days of culture were dark-adapted for 30 min. The saturation light impulse 7,500 µmol m^−2^ s^−1^ and actinic light 3,000 µmol m^−2^ s^−1^ were used. Using fluorescence parameters: the minimum chlorophyll fluorescence (*F*_0_), the maximum chlorophyll fluorescence (*F*_m_) and variable fluorescence (*F*_v_), the following was calculated: the maximal efficiency of PSII in the dark-adapted state (*F*v/*F*m) (Krause and Weis 1991). Maximum primary yield of photochemistry of PSII was determined as the ratio of *F*v to *F*_0_ (*F*v/*F*o). Experiments were repeated 3 times in 3 independent replications.

### RuBisCO small and large subunits determination

#### Isolation of RuBisCO for SDS-PAGE separation

Isolated (separately upper and lower) cotyledons of 5-, 8-, and 10-day-old control and NO-treated seedlings were homogenized in 0.1 M Tris–HCl (pH 8.0) buffer containing 1 mM EDTA, 2 % (w/v) polyvinylpolypyrrolidone (PVPP), 5 mM dithiothreitol (DTT), 5 mM MgCl_2_, 0.1 % (w/v) protease inhibitor cocktail (Sigma-Aldrich P9599), 10 % (v/v) glycerol, and 0.1 % (w/v) Tween 20 in ice bath. After centrifugation (MPW centrifuge, Poland) at 15,000*g* for 10 min at 4 °C supernatant was passed through the nylon net and collected for further analyses.

#### Western blotting analysis of RuBisCO subunits

For Western blotting analysis of RuBisCO subunits, protein extracts from cotyledons were suspended in 63 mM Tris–HCl electrophoresis buffer, pH 6.8, 1 % (w/v) SDS, 10 % (v/v) glycerol and 0.01 % (w/v) bromophenol blue, 20 mM DTT and incubated at 95 °C for 5 min. For immunoblotting 20 µg of total proteins were loaded per line and separated on 12.5 % standard SDS-polyacrylamide gels (SDS-PAGE) according to Laemmli (1970). After SDS-PAGE proteins were electrotransferred to nitrocellulose membranes (Pure Nitrocellulose Membrane, Z670979, Sigma-Aldrich) according to Towbin et al. (1979) using a Bio-Rad wet electroblotting system. The membranes after transfer were stained for protein visualization using 0.2 % w/v Ponceau Red in 2 % 9 v/v acetic acid solution. Nitrocellulose membranes were blocked overnight at 4 °C with 5 % (w/v) non-fat dry milk. Immunolabelling of RuBisCO small and large subunits was done separately. Western blotting was carried out by incubation of the membranes with the primary antibodies Rbcl (RuBisCO large subunit Agrisera AS03 037) or Rbcs (RuBisCO small subunit Agrisera AS03 259) at dilution 1:5000 each. Secondary antibodies anti-rabbit (Agrisera AS09 607) were conjugated with alkaline phosphatase and used at dilution 1:8000. Visualization of RuBisCO small or large subunits was performed using a mixture of 0.2 mM nitroblue tetrazolium salt (NBT) and 0.21 mM 5-bromo-4-chloro-3-indolyl phosphate (BCIP) in buffer 100 mM Tris–HCl pH 9.5, 100 mM NaCl, 5 mM MgCl_2_. Assays were done in 2–3 independent experiments and their typical results are shown.

### Protein determination

Protein concentration was measured according to Bradford (1976) using bovine serum albumin (BSA) as a standard.

### Sugars concentration measurement

Cotyledons, separately (upper and lower) isolated from control and NO-treated seedlings after 5, 8 and 10 days of culture were collected and used for reduced sugars determination with copper-2,2′-bicinchonic acid (BCA) reagent (Waffenschmidt and Jänicke 1987). Plant material (0.2 g) was placed in 2 ml of 50 % ethanol and homogenized at room temperature. After centrifugation (MPW-350R centrifuge, 10,000*g*, 10 min) supernatant was collected and used for glucose analysis. Determination was done after 15 min incubation at 100 °C using Hitachi U-2900, Japan spectrophotometer at 560 nm. Assays were done in 3–4 independent experiments, with 3 repetitions. The results were expressed as glucose (Glc) µmol mg^−1^ FW.

### Ultrastructural analysis with the transmission electron microscopy

Upper cotyledons isolated separately from seedlings after 5, 8 and 10 days of the culture were cut into 0.5 mm pieces and immediately immersed in a fixative composed of 2 % (w/v) paraformaldehyde and 2 % (v/v) glutaraldehyde in 0.05 M sodium cacodylate buffer (pH 7.2; Sigma) (Karnovsky 1965) for 2 h at room temperature. The probes were post-fixed in 2 % (w/v) osmium tetroxide in 0.05 M cacodylate buffer for 2 h at 4 °C, dehydrated in ethanol and propylene oxide and embedded in EPON epoxy resin (Epon 812, Sigma). Ultrathin (70–80 nm) sections of the polymerized probes were taken on a UCT ultramicrotome (Leica Microsystems) and mounted on formvar-coated single-slot copper grids. Sections were stained with 1.2 % ethanolic uranyl acetate and 2.5 % lead citrate and examined with a 268D Morgagni transmission electron microscope (FEI, Hillsboro, OR, USA) operating at 80 kV. The images were taken with a Morada digital camera (Olympus SIS, Münster, Germany) at 10 M pix resolution.

### Statistics

Data were analyzed using the StatGraphics 5.1; mean ± SE was computed for each experiment and significance of differences was assessed with Tukey’s studentized range test or Student’s *t* test. Differences are considered significant at *P* ≤ 0.05.

## Results

### Growth and development of seedlings

**Fig. 2:**
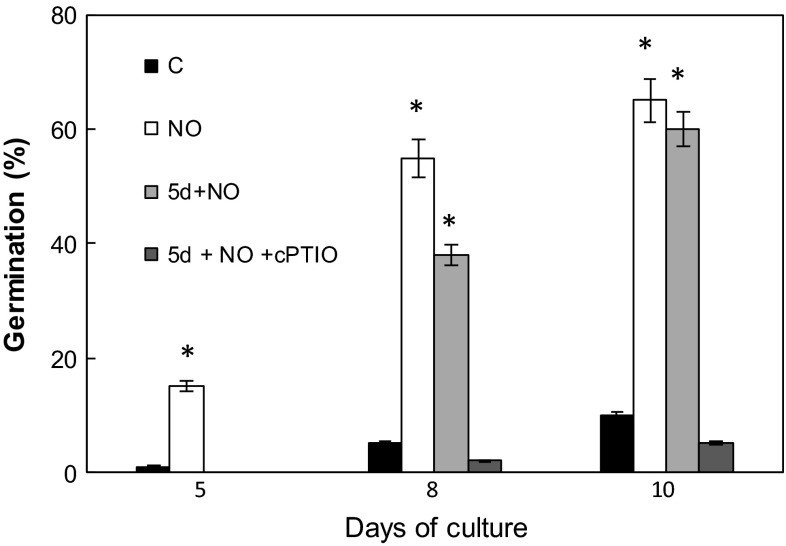
Germination (%) of the control embryos (C), embryos shortly treated with NO after imbibition (NO) or after 5 days of culture (5d+NO) and embryos (5d+NO) imbibed in 0.3 mM cPTIO (5d+NO+cPTIO). Germination was counted as  % of embryos with radicals of 2–3 mm long and exhibiting typical gravitropic bending. *Values* are average ± SE of at least 4 replicated experiments. *Asterisk* significance from control at the same time of culture period at *P* ≤ 0.05

**Fig. 3:**
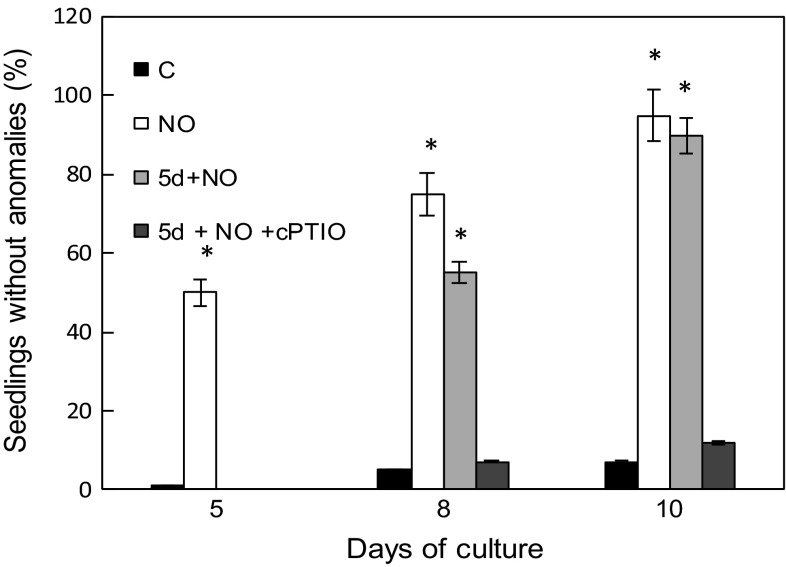
Development of normal seedlings (without any anomalies defined as asymmetric growth and greening of cotyledons) (%) growing from control embryos (C) and embryos shortly treated with NO after imbibition (NO) or after 5 days of culture (5d+NO) and application of 0.3 mM cPTIO (5d + NO + cPTIO). Values are average ± SE of at least 4 replicated experiments. *Asterisk* significance from control at the same time of culture period at *P* ≤ 0.05

As reported in our previous papers (Gniazdowska et al. 2010a, b) embryos isolated from dormant apple seeds did not germinate, and exhibited typical malformations in growth and greening of cotyledons. Short-term pre-treatment of dormant embryos with acidified nitrite (NO) at the beginning of experiment (after seed coat removal) resulted in stimulation of germination and led to development of seedlings with two green cotyledons of equal size (Figs. 1, 2, 3). The morphological effect of embryo treatment with SNAP (5d+SNAP) or vapors of acidified nitrite (5d+NO) was the same (Fig. 1), so in further experiments only acidified nitrite (NO) was used as NO source. Short-term treatment with NO or SNAP of 5-day-old embryos with malformations (5d+NO) or (5d+SNAP), produced a similar effect, as observed after treatment of dormant embryos with NO at once after removal of the seed coat (NO) (Figs. 1, 2 and 3). As a result, independent of the timing of NO application the equal amount of seedlings with uniform, two greening cotyledons were detected after 10 days of culture (Figs. 1 and 3). After short treatment with NO of 5-day-old dormant embryos (5d+NO), showing the typical developmental anomalies, germination was observed without any delay (Figs. 1 and 2). In the prolonged experiment (after 8 days of culture), germination of these embryos (5d+NO) was only about 25 % lower as compared to the germination of embryos that have been treated with NO just after removal of seed coverings (Fig. 2). On the 10th day of culture the embryos treated with NO at the age of 5 days (5d+NO) germinated in 58 %, while the embryos pre-treated with NO at once after removal of the seed coat layers (NO) germinated in 67 % (Fig. 2). Application of cPTIO strengthened dormant state of embryos (5d+NO+cPTIO) (Fig. 2), resulting in inhibition of development of seedling without anomalies (Fig. 3).

### Hydrogen peroxide concentration in cotyledons of developing seedlings

**Fig. 4:**
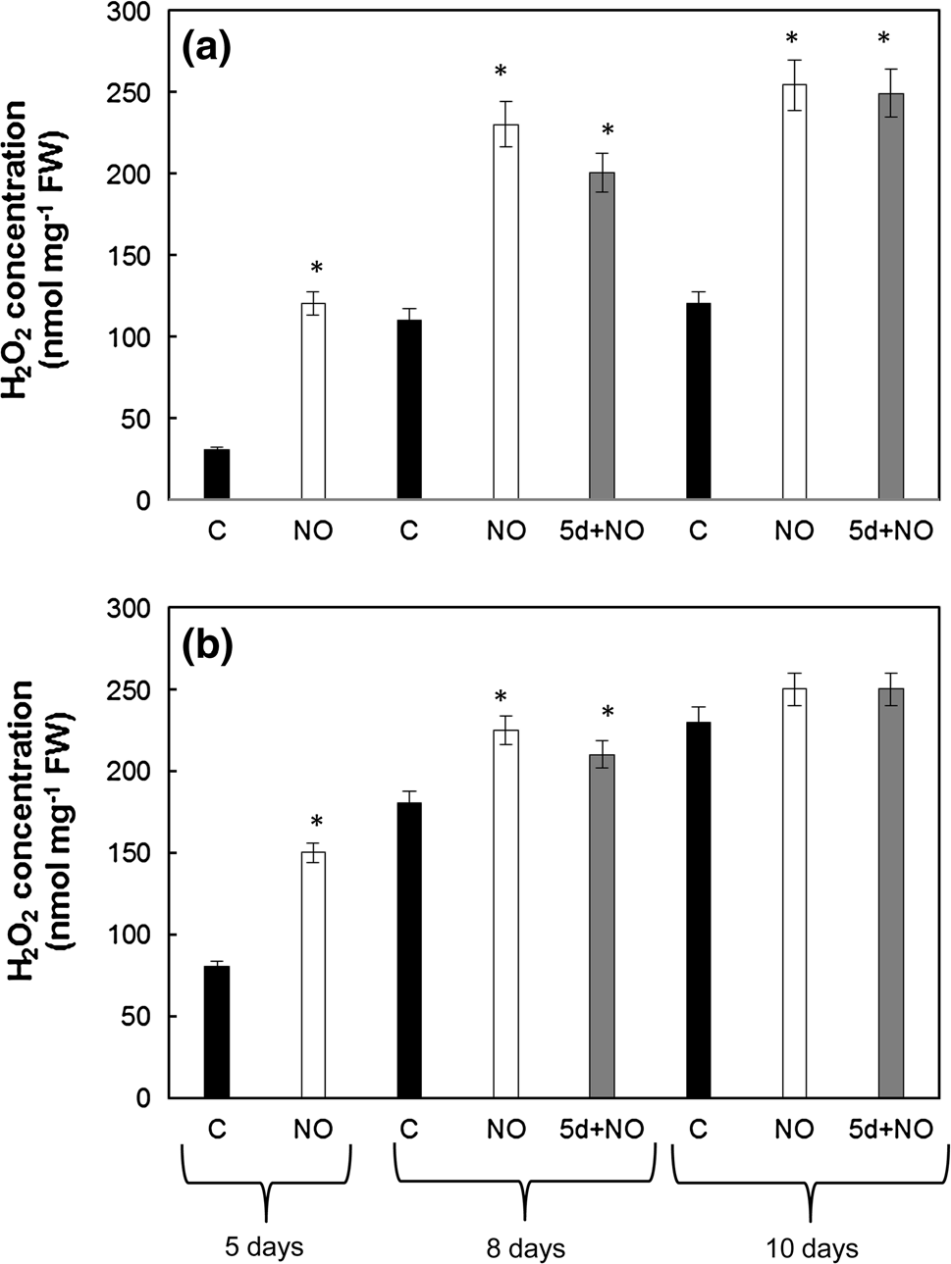
Concentration of H_2_O_2_ in upper (**a**) and lower (**b**) cotyledons of 5-day-old control seedlings (C), seedlings developed from embryos shortly treated with NO after imbibition (NO) after 5, 8 and 10 days of culture or control seedlings shortly treated with NO after 5 days of culture (5d+NO) determined after 8 and 10 days of culture. Values are average ± SE of 4–5 replicated experiments. *Asterisk* significance from control at the same time of culture period at *P* ≤ 0.05

The lowest concentration of H_2_O_2_ was observed in upper, white cotyledons of dormant embryos at the 5th day of culture (C) (Fig. 4a, b). Concentration of H_2_O_2_ in the upper cotyledons of control embryos increased during the culture period to the value of around 120 nmol mg^−1^ FW. After 8 and 10 days of culture, lower cotyledons of control embryos (C) and both cotyledons of seedlings developing from embryos treated with NO (NO and 5d+NO) contained similar concentration (about 230–250 nmol mg^−1^ FW) of H_2_O_2_ (Fig. 4a, b). At the point of termination of the experiment, the ratio relating to the concentration of H_2_O_2_ in the upper cotyledon to H_2_O_2_ concentration in the lower cotyledon in seedlings growing from NO-treated embryos (NO or 5d+NO) reached the value 1.0, whereas the ratio in dormant embryos was 0.5.

### Chlorophyll concentration in cotyledons of developing seedlings

During the culture, an increase in the concentration of total chlorophyll (chl *a* + *b*) in the cotyledons of all tested seedlings or embryos was detected (Table 1). In the cotyledons of 8-day-old seedlings growing from the NO embryos (pre-treated with NO at the beginning of experiment) concentration of total chl *a* + *b* increased fourfold as compared to day 5, while in the 5d+NO seedlings the chl *a* + *b* concentration increased about tenfold in comparison to dormant ones. The highest concentration of chl *a* + *b* was recorded at the 10th day in the cotyledons of seedlings growing from embryos treated with NO (both NO and 5d+NO). The cotyledons of seedling developing from embryos treated with NO independently of the point of treatment (both NO and 5d+NO) after 10 days of the culture contained a similar amount of chl *a* + *b* reaching the value about 0.25–0.29 mg g^−1^ FW. The lowest concentration of total chlorophyll characterized upper cotyledons of control embryos. Upper cotyledons of control embryos remained white till the termination of experiment. In the opposite, the concentration of chl *a* + *b* in the lower cotyledons of these embryos was high. It was similar to the concentration determined in the lower cotyledons of embryos (5d+NO), with morphological malformations removed by delayed NO treatment at 5th day of culture, and similar to chl *a* + *b* concentration in lower cotyledons of seedling developing from NO pre-treated embryos.

**Table 1:** Chlorophyll *a* and chlorophyll *b* concentration (mg g^−1^ FW) in upper (U) and lower (L) cotyledons of embryos or seedlings developing from control dormant embryos, embryos pre-treated with NO immediately after seed coat removal (NO), and embryos fumigated with NO after 5 days of imbibition in water (5d+NO). Chlorophyll concentration was determined at 5th, 8th and 10th day of culture period

Days of culture	Treatment	Cotyledon	Chl *a*	Chl *b*	Chl *a* + *b*	Ratio chl *a*:chl *b*
5	Control	U	0.0	0.0	0.0	–
		L	0.0130 ± 0.0020	0.0	0.0120 ± 0.0040	–
	NO	U	0.0230 ± 0.0020*	0.0080 ± 0.0021*	0.0300 ± 0.0030*	2.8
		L	0.0250 ± 0.0020	0.0070 ± 0.0018*	0.0310 ± 0.0050*	3.5
8	Control	U	0.0016 ± 0.0005	0.0002 ± 0.0001	0.0016 ± 0.0005	8.0
		L	0.0420 ± 0.0060	0.0085 ± 0.0012	0.0420 ± 0.0070	4.9
	NO	U	0.1270 ± 0.0320*	0.0190 ± 0.0023*	0.1200 ± 0.0200*	6.7
		L	0.1170 ± 0.0230*	0.0270 ± 0.0056*	0.1200 ± 0.0300*	6.3
	5d+NO	U	0.0490 ± 0.0080*	0.0073 ± 0.0010	0.0550 ± 0.0100	6.7
		L	0.0510 ± 0.0090	0.0270 ± 0.0060*	0.0750 ± 0.0120*	1.9
10	Control	U	0.0110 ± 0.0020	0.0	0.0100 ± 0.0020	–
		L	0.1190 ± 0.0350	0.0125 ± 0.0020	0.1250 ± 0.0300	9.5
	NO	U	0.2450 ± 0.0650*	0.0400 ± 0.0070*	0.2700 ± 0.0700*	6.2
		L	0.2720 ± 0.0780*	0.0400 ± 0.0040*	0.2900 ± 0.0800*	6.8
	5d + NO	U	0.2300 ± 0.0560*	0.0510 ± 0.0100*	0.2700 ± 0.0650*	4.5
		L	0.2250 ± 0.0490*	0.0290 ± 0.0030*	0.2450 ± 0.0500*	7.7

Values are average ± SE of 4–5 replicated experiments

Asterisk (*) indicates significance from control at the same time of culture at *P* ≤ 0.05

Analysis of chl *a* and separately chl *b* indicated higher concentration of chl *a* as compared to chl *b* in all tested plants (Table 1). In lower cotyledons of dormant embryos, the ratio chl *a*:chl *b* increased during the culture to about 5 and 9.5 at 8th and 10th day, respectively. In upper cotyledons of dormant embryos only chl *a* has been noticed at measurable level, as negligible amount of chl *b* was detected (Table 1). In seedlings developing from NO-treated dormant embryos (NO), chl *a*:chl *b* ratio in both cotyledons elevated from around 3 (noted at the 5th day) to 6.2–6.8 after 10 days of culture. In 10-day-old seedlings obtained by NO treatment of abnormal embryos (5d+NO) chl *a*:chl *b* ratio differed in upper and lower cotyledons, and was about twice higher in lower one, which was green at the moment of NO application. In general, NO treatment increased concentration of both chl *a* and chl *b* predominantly in upper cotyledons, although in lower cotyledons of NO-stimulated seedlings chlorophyll content was doubled as compared to lower cotyledons of control seedlings (Table 1).

### Photosynthetic activity of developing seedlings

**Fig. 5:**
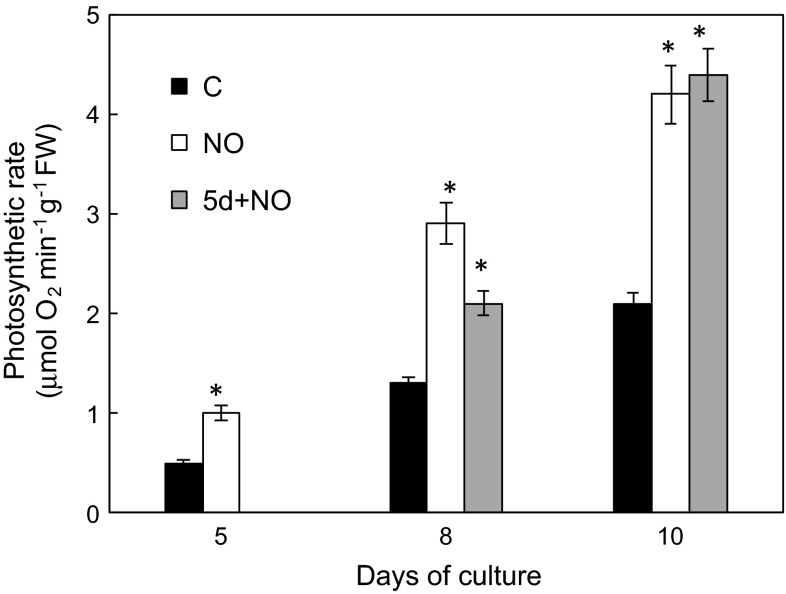
Photosynthetic activity of control seedlings (C), after 5, 8 and 10 days of culture, and seedlings developed from embryos shortly treated with NO after imbibition (NO) after 5, 8 and 10 days of culture or shortly treated with NO control seedlings after 5 days of culture (5d+NO) and after 8 and 10 days of culture. Values are average ± SE of 3–4 replicated experiments. *Asterisk* significance from control at the same time of culture period at *P* ≤ 0.05

Photosynthetic activity of intact seedlings was determined as O_2_ evolution and chlorophyll *a* fluorescence. Net photosynthetic rate of 10-day-old control seedlings increased around fourfold in comparison to its value on the 5th day (Fig. 5). NO short-term treatment of dormant embryos resulted in stimulation of photosynthetic activity, which was twice higher in 5-day-old NO seedlings than in control. Such stimulation was constant during the whole culture period. Delayed treatment of seedlings with anatomical anomalies (5d+NO) with NO led to rapid stimulation of photosynthetic activity. In 8-day-old (5d+NO) seedlings it was only 20 % lower than in NO seedlings, and increased during next 2 days achieving a value of about 4.5 μmol min^−1^g^−1^FW, which was twice higher than that one observed for control seedlings (Fig. 5).

### Fluorescence of chlorophyll a

The maximum photochemical efficiency of PSII was determined from the ratio of variable fluorescence (*F*v) to maximum fluorescence (*F*m). While maximum primary yield of photochemistry of photosystem II was determined as the ratio of Fv to minimal fluorescence in dark-adapted state (*F*_0_) (*F*v/*F*_0_), *F*v/*F*m in lower cotyledons of NO-treated seedlings (NO and 5d+NO) was slightly increased compared to control only at the beginning of the experiment (Table 2). In upper cotyledons till the 10th day it was measurable only in NO-treated plants. Its value increased constantly, but during the entire period of experiment did not reach its typical value (0.8).

**Table 2:** Chlorophyll *a* fluorescence parameters in upper and lower cotyledons of embryos or seedlings developing from control dormant embryos, embryos pre-treated with NO immediately after seed coat removal (NO), and embryos fumigated with NO after 5 days of imbibition in water (5d+NO)

Culture period	Treatment	Cotyledon	*F*v/*F*m	*F*v/*F*_0_
5 days	Control	U	nd	nd
		L	0.43 ± 0.03	0.80 ± 0.04
	NO	U	0.61 ± 0.07*	1.77 ± 0.30*
		L	0.58 ± 0.06	1.50 ± 0.20*
8 days	Control	U	nd	nd
		L	0.48 ± 0.06	0.90 ± 0.10
	NO	U	0.76 ± 0.05*	3.23 ± 0.70*
		L	0.75 ± 0.05	3.20 ± 0.50*
	5d+NO	U	0.49 ± 0.06*	1.29 ± 0.20*
		L	0.54 ± 0.05	1.22 ± 0.20
10 days	Control	U	0.04 ± 0.01	0.05 ± 0.01
		L	0.56 ± 0.07	1.70 ± 0.30
	NO	U	0.55 ± 0.08*	1.23 ± 0.10*
		L	0.55 ± 0.07	1.30 ± 0.10
	5d+NO	U	0.60 ± 0.10*	1.55 ± 0.20*
		L	0.55 ± 0.07	1.30 ± 0.20

Fluorescence was determined at 5th, 8th and 10th day of culture period. Values are mean ± SE of 3 experiments, *nd* not detected

Asterisk (*) indicates significance from control at the same time of culture at *P* ≤ 0.05

The level of maximum primary yield of photochemistry of PSII (Fv/F_0_) was affected by NO application (Table 2). It was measurable only in greening cotyledons, so was not detected in upper white cotyledons of dormant embryos. In lower, getting green cotyledon of dormant embryos *F*v/*F*_0_ increased constantly during culture period and at the end of experiment was doubled in comparison to its initial rate. In both lower and upper cotyledons of NO seedlings, Fv/F_0_ value increased transiently at the 8th day of culture and declined in 10-day-old seedling to the level observed at the beginning of experiment. Similarly, in 5d+NO seedlings *F*v/*F*_0_ rose rapidly in both cotyledons after NO treatment and remained constant till the 10th day of culture.

### Amount of RuBisCO large and small subunits in cotyledons of developing seedlings

**Fig. 6:**
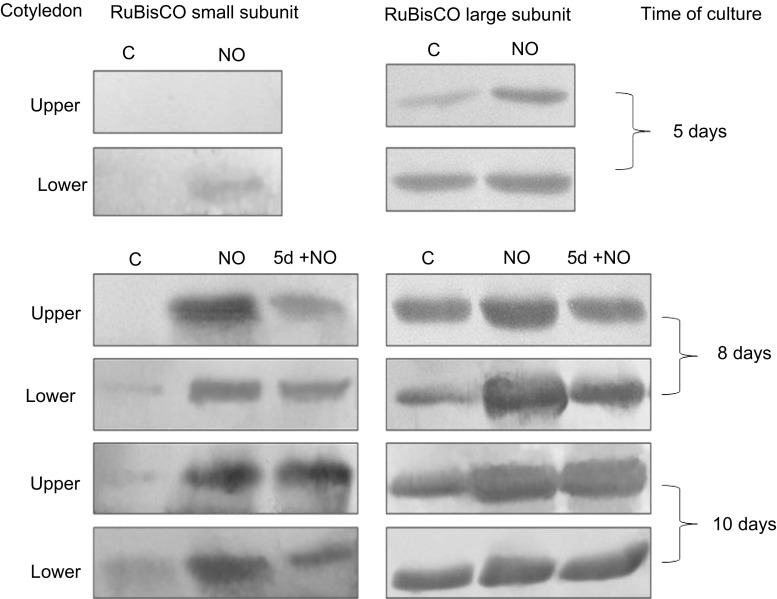
Western blot analysis of RuBisCO large and small subunits in upper and lower cotyledons of 5-, 8-, 10-day-old control seedlings (C), cotyledons isolated from seedlings developed from embryos shortly treated with NO (NO) after 5, 8 and 10 days of culture, and cotyledons isolated from control seedlings treated with NO after 5 days of culture (5 + NO) after 8 and 10 days of experiment. Typical blots are shown

In the upper cotyledons of dormant embryos there was no band indicating the presence of small RuBisCO subunit (Fig. 6). Short-term treatment with NO of dormant apple embryos or 5-day-old abnormal seedlings resulted in appearance of small subunit of RuBisCO in the upper cotyledons of 8- and 10-day-old seedlings. In lower cotyledons NO led to enhanced accumulation of this subunit independent of timing of NO application.

In contrast, NO treatment did not induce significant modification in content of the large RuBisCO subunit in both cotyledons of developing seedlings, although a higher than in control amount of large RuBisCo subunit was noticed mainly in lower cotyledons of 8-day-old NO seedlings (Fig. 6).

### Sugar content in cotyledons of developing seedlings

**Fig. 7:**
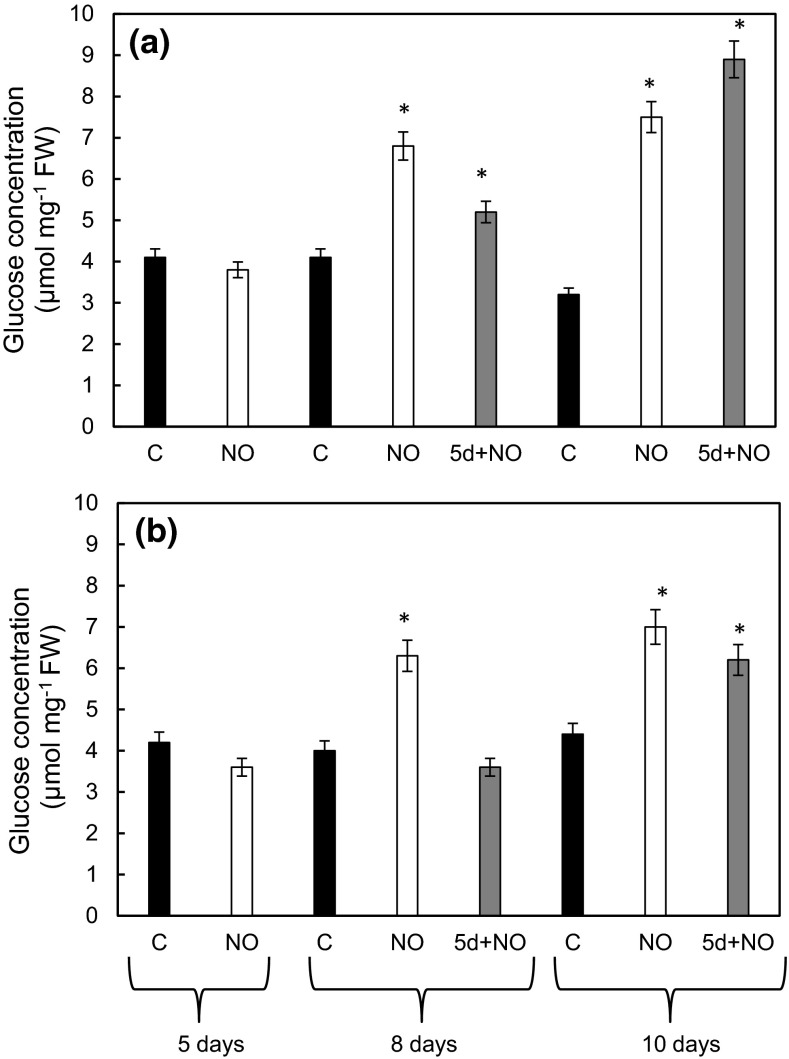
Concentration of Glc in upper (**a**) and lower (**b**) cotyledons of 5-, 8- and 10-day-old control seedlings (C), seedlings developed from embryos shortly treated with NO after imbibition (NO) after 5, 8 and 10 days or seedlings developed from 5-day-old control seedlings shortly treated with NO (5d+NO) after 8 and 10 days of culture. Values are mean ± SE of at least 3 replicated experiments. *Asterisk* significance from control at the same time of culture period at *P* ≤ 0.05

Glucose (Glc) concentration in cotyledons of control seedlings was stable during culture period (Fig. 7a, b) It was similar in both upper and lower cotyledons and reached a value of about 3.5–4.2 µmol mg^−1^FW. NO treatment enriched glucose concentration in the same extent in upper and lower cotyledons. In seedlings developed from NO pre-treated embryos after 10 days of culture Glc was doubled as compared to its concentration in 5-day-old embryos. Delayed NO treatment of embryos with morphological anomalies resulted in accumulation of Glc in cotyledons. Its concentration was threefold higher than in control in the upper cotyledons and 50 % elevated in lower ones (Fig. 7a, b).

### Plastids ultrastructure

**Fig. 8:**
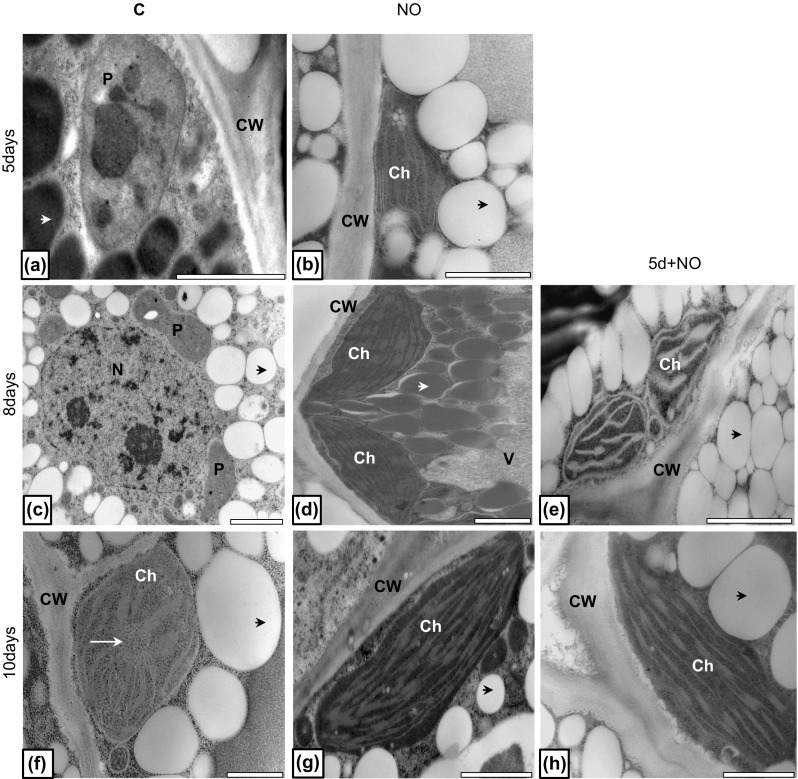
Ultrastructure of cells of upper cotyledon of control (**a**, **c**, **f**), NO-treated embryos (**b**, **d**, **g**) and plantlets developed from abnormal embryos treated with NO after 5 days of growth (**e**, **h**). Photographs were made after 5 (**a**, **b**), 8 (**c**, **d**, **e**) and 10 (**f**, **g**, **h**) days of culture. Micrographs are representative for ultrastructure of upper cotyledons of embryos after each treatment and specified period of culture. *Black arrow* cytoplasmic domain rich in lipid bodies; *white short arrow* cytoplasmic protein bodies; *white long arrow* prolamellar body, *CW* cell wall, *P* proplastid, *Ch* chloroplast, *V* vacuole. *Bar* 2 µm (**a**, **c**–**e**, **g**), 1 µm (**b**, **f**, **h**)

Upper cotyledons of 5-day-old control seedlings were characterized by presence of proplastids (Fig. 8a). Developmental arrest of proplastids in upper cotyledons of dormant embryos was observed until 8th day of the culture (Fig. 8c). At the 10th day of culture of control embryos chloroplasts at early stage of maturity, with well-visible prolamellar body were detected (Fig. 8f). Upper cotyledons of seedlings developed from embryos treated with NO (3 h) after 5 days of culture had chloroplasts rather than proplastids with developed lamellar system (Fig. 8b). From 8th day of the culture in upper cotyledons of NO-treated embryos fully developed chloroplasts were observed (Fig. 8d, g). Short-term treatment of control abnormal 5-day-old embryos led to stimulation of chloroplast biogenesis after 8th day of the culture (Fig. 8e). Fully developed lamellar system was observed in cotyledons of these plants after 10th day of the culture (Fig. 8g, h).

## Discussion

Seedlings growing from embryos of dormant apple seeds are characterized by the presence of only one well-developed cotyledon, while the another (upper) one remains of small size and white in color even after prolonged culture in light (Lewak 2011). These anomalies are not observed in seedlings originating from embryos shortly treated with various NO donors (SNP, SNAP) just immediately after isolation from seed coats (Gniazdowska et al. 2010a, b). In this work it has been shown that short-term treatment with NO or SNAP of 5-day-old abnormal seedlings led to reverse of morphological abnormalities and this effect was similar to that one observed after treatment with 3 mM SNAP or 5 mM SNP as reported also in our previous experiments (Gniazdowska et al. 2010a, b). Moreover, the pivotal role of NO in this process was confirmed using NO scavenger. Abnormal seedlings shortly treated with NO and placed in cPTIO solution were the same in morphology as control, with shortened embryonic axis and abnormal cotyledons. Physiological development of cotyledons is connected with the progression of photosynthetic activity. The first visible symptom of initiation of autotrophy by NO treatment was greening of cotyledons in developing seedlings. After embryos exposition to NO, increase of chlorophyll concentrations was observed in both (upper and lower) cotyledons and this reaction was independent of the timing of NO application. A similar pattern of greening of the cotyledons was demonstrated in other experiments concerning treatment with hydrogen cyanide (HCN). Short-term pre-treatment of dormant apple embryos with HCN resulted in visible and fast greening of cotyledons (Gniazdowska et al. 2010b). This toxic molecule is naturally produced in apple embryos and released inside the cells as a result of degradation of cyanogenic compounds that occurs during dormancy loss by cold stratification (Lewak 2011 and citation therein). Release of HCN in cold-stratified apple seeds is accompanied by increased NO emission from embryonic axis (Dębska et al. 2013). Taking into account the similarities of HCN and NO impact on cotyledons greening we can assume the cross-talk between both molecules during seedling development.

NO and ROS, including H_2_O_2_, are molecules of bimodal function, depending on concentration. They influence each others’ production and concentration, thus affect redox status of the tissues (Galvez-Valdivieso and Mullineaux 2010; Yu et al. 2014). Our former results indicated involvement of short-term (3 h) application of H_2_O_2_ (1 mM) in equal greening of apple cotyledons (Gniazdowska et al. 2010b). We also presented that short-term pre-treatment of dormant embryos with NO stimulated H_2_O_2_ production leading to overcome dormancy (Gniazdowska et al. 2010b; Krasuska et al. 2014). Data shown in this work indicated that increase in H_2_O_2_ level in the tissue correlated with greening of the cotyledons, stimulated by NO application. At the third day after NO treatment of 5-day-old control seedlings (5d+NO) H_2_O_2_ concentration in cotyledons was almost twice higher in upper one as compared to the control. It arises the question whether the observed enlargement in ROS is the result of greening of cotyledons and activation of photosynthetic electron transport chain or in opposite, is a stimulus of this process. It is believed that products of ROS reaction with lipids and/or proteins could also act as compounds involved in chloroplast-to-nucleus signaling (retrograde signaling). ROS-mediated peroxidation of polyunsaturated fatty acids leads to formation of cyclic oxylipins, a potent inducer of nuclear gene expression (Galvez-Valdivieso and Mullineaux 2010). Protein modification via ROS leads to production of carbonylated peptides which can be considered as specific (organelle specific) signals transported to nucleus (Møller and Sweetlove 2010). Our previously presented results indicated involvement of NO in protein oxidation. NO treatment of dormant apple embryos resulted in decline in the level of protein carbonyl groups as germination was prolonged (Krasuska et al. 2014). Thus, the participation of such modified proteins in retrograde signaling could be possible, but needs further verification. Modification of redox state (by ROS) is proposed as one of the mechanisms of regulation of chlorophyll biosynthesis (Stenbaek and Jensen 2010). Some of the chloroplast-localized enzymes of Calvin cycle or starch biosynthesis are under redox regulation (Geigenberger et al. 2005). On the other hand, NO can reduce symptoms of oxidative stress by induction of the defense mechanisms. Pre-treatment of tall fescue (*Festuca arundinacea*) leaves with SNP lowered light-induced electrolyte leakage and malondialdehyde concentration. Moreover, activities of enzymes of cellular ROS-modulating system were stimulated (Xu et al. 2010).

The data presented by Beligni and Lamattina (2000) on etiolated wheat (*Triticum aestivum* L.) seedlings confirmed that NO is involved in the regulation of biosynthesis of chlorophyll. Light-mediated chlorophyll accumulation of barley (*Hordeum vulgare* L.) seedlings was also shown after SNP treatment and confirmed using PTIO (Zhang et al. 2006). Although, these data are doubtful as authors treated plants with NO donors (including also SNP) in darkness or dim green safe light, not sufficient for SNP decomposition, and illuminated seedlings only after treatment. Experiments carried out on wheat seedlings treated with 100 µM SNP and grown on medium containing various concentrations of iron (Fe), showed that NO not only affected the uptake and binding of this microelement, but also prevented chlorosis. Protective effect of NO on Fe deficiency was associated with stimulation of the conversion of Mg-protoporphyrin to chlorophyllide, then the chlorophyll *a* and *b* (Abdel-Kader 2007). This effect was reversed after the application of the 100 mM methylene blue, used as inhibitor of guanyl cyclase (enzyme acting in NO signaling pathway). It suggests that NO is involved in the biosynthesis of chlorophyll and may contribute to specific steps of this process. As the culture of apple embryos was carried out some changes in the chlorophyll content were observed. The chlorophyll concentration in the upper cotyledons of control, 5-day-old plants was very low, undetectable by the method used. NO treatment of these seedlings led to huge increase in chlorophyll *a* in upper cotyledons. Moreover, chlorophyll *b* concentration in lower cotyledons of NO-treated seedlings was detected at around 3 times higher level than in control. After 10 days of culture, NO-treated seedlings (5d+NO) were characterized by almost the same amount of chlorophyll *a* and *b* as compared to seedlings developed from NO pre-treated embryos. In addition, upper cotyledons of developing seedlings (5d+NO) were greening faster than the upper cotyledons of seedlings grown from embryos treated with NO just after removal of seed coats. Short-term treatment of 5-day-old control seedlings with NO did not disturb chlorophyll biosynthesis in lower cotyledons. Similar observations by Zhang et al. (2006) showed an increase in NO production in parallel to the greening of barley seedlings. These changes were accompanied by the development of the thylakoids in chloroplasts. Linking the results obtained in our study and observations of Zhang et al. (2006) we can assume that more rapid greening of the upper cotyledons of seedlings treated with NO at the stage of young, abnormal seedling (5d+NO) than coloration of cotyledons of seedlings growing from embryos shortly pre-treated with NO (NO) is due to longevity of light exposure. It was reported that in yeast, light increased nitrite-dependent NO synthesis (Ball et al. 2011). However, light-stimulated NO production in apple cotyledons needs to be proved by further studies.

Our findings also led to the assumption that NO acts as a member of the light-induced signaling cascade. Light intensity, quality and duration govern dark-to-light transition that occurs in the post-germination ontogeny (switch from heterotrophy to autotrophy). This process is under control of phytochromes and cryptochromes. Using NO-deficient mutants of Arabidopsis and mutants with increased endogenous NO levels, as well as NO donor (SNP), Lozano-Juste and León (2011) indicated NO involvement in photomorphogenesis. In addition, they suggested NO action downstream of phytochrome B in red light signaling. Short-term treatment with NO increased the rate of photosynthesis of apple seedlings grown either from both dormant embryos or 5-day-old dormant abnormal seedlings. Short treatment with NO of control seedlings (analyzed at long-term perspective) is necessary for transition from heterotrophy to autotrophy. High photosynthetic activity in cotyledons of apple seedlings was observed previously in plants that underwent dormancy loss by cold stratification (Lewak 2011 and citation therein).

NO binds reversibly to the several sites in photosystem II (PSII), slowing down electron transport (Wodala et al. 2008). Inhibition of light-dependent reaction can be estimated by parameters of chlorophyll *a* fluorescence. Thus, we measured chlorophyll *a* fluorescence and calculated its basic parameters *F*_v_/*F*_m_ and *F*_v_/*F*_0_ in cotyledons. Their values differed from typical rates observed under most frequently analyzed stressors (drought or salinity). Maximum quantum yield of PSII (*F*_v_/*F*_m_) ratio is considered to describe the effectiveness of the PSII in the primary photochemical reactions. It is proportional to the quantum yield of the photochemical phase of photosynthesis. Any decrease in *F*_v_/*F*_m_ indicates that PSII suffers from damage and that the key reactions of photosynthesis are inhibited (Baker 2008). The value of *F*_v_/*F*_m_ parameter in leaves under non-stress condition should reach the value around 0.8 suggesting that the light phase of photosynthesis occurs efficiently. Its value increased in the upper cotyledons of seedlings treated with NO but not immediately after treatment. In pea (*Pisum sativum* L.) leaves incubation in 1 mM nitrosoglutathione (GSNO) for 2 h resulted in reduction of F_v_/F_m_ rate (Wodala et al. 2008). On the other hand, treatment with SNAP of the isolated chloroplasts did not affect *F*_v_/*F*_m_ (Takahashi and Yamasaki 2002). In our study, the enlargement in chlorophyll content in the upper cotyledons of seedlings developing after NO treatment was accompanied by an increase in the *F*_v_/*F*_0_ ratio. Similar results were reported for barley seedlings after SNP application. However, treatment of the plants with NOS inhibitor N-Omega-nitro-l-arginine (l-NAA) or PTIO led to opposite effect. Decline in NO concentration due to l-NAA or PTIO was associated with reduced value of *F*_v_/*F*_m_. The authors suggested that NO may be involved in the formation of functional PSII (Zhang et al. 2006). So, we can assume that short-term treatment of dormant embryos or abnormal seedlings with NO is not destructive, but positively modifies the efficiency of processes associated with the light phase of photosynthesis.

Functional (active) RuBisCO (EC 4.1.1.39) protein of higher plants consists of eight small subunits, and eight large subunits (Suzuki et al. 2010 and citation therein). NO short-term treatment of apple embryos increased amount of small subunit of RuBisCO. Dormant embryos (control) were deficient with small subunit of RuBisCO in the upper cotyledons until the 10th day of culture and until the 8th day in the lower ones, getting green. After treatment with NO of abnormal embryos (5d+NO) abundance of RuBisCO small subunit increased. No decline in accumulation of large subunit of RuBisCO was noticed. At the same period large subunit was present in cotyledons of both control seedlings and NO-treated ones (NO and 5d+NO). These data suggest that dormancy of apple embryos, defined as formation of abnormal seedlings, may be related to disturbances in light perception and signaling. This “blindness” of dormant embryos could be broken by NO. It seems also that synthesis of small subunit in cotyledons occurs after seed dormancy lost. When chloroplast biogenesis is blocked, photosynthesis-associated nuclear genes’ (PhANGs) expression (including small subunit of RuBisCO) is repressed (Ruckle et al. 2007). Appearance of small subunit of RuBisCO after NO treatment suggests that its synthesis depends on retrograde signaling (from chloroplast-to-nucleus) with NO acting as a key agent. Non-parallel pattern of appearance of small and large RuBisCO subunits, although unexpected and uncommon could be explained by storage of long-lived mRNA or proteins in seeds. We could suspect that mRNA or protein of large subunit could be stored in cells and its transcription or translation in control seedlings is not affected (do not depend on NO). In the contrary, no mRNA of small RuBisCO subunit is stored in cotyledons of dormant embryos. It could be possible, as although synthesis of new transcripts was necessary during imbibition of non-dormant Arabidopsis seeds, α-amanitin (an inhibitor of eukaryotic mRNA synthesis) did not block germination (Rajjou et al. 2004), demonstrating the existence of long-lived stored mRNA, utilized during germination or early growth of seedlings. In addition, treatment of pea (*Pisum sativum*) seedling with α-amanitin indicated that steady-state level of RuBisCO large subunit mRNA is not directly affected by mRNA of small subunit (Sasaki 1986). The authors suggested also that light induction of transcription of RuBisCO small subunit mRNA was not required for large subunit mRNA transcription.

Experiments carried out on maize (*Zea mays* L.) plants demonstrated NO impact on transcription of genes coding the large subunit of RuBisCO (Graziano et al. 2002). Abat et al. (2008) showed that NO treatment of kataka-taka (*Kalanchoe pinata*) resulted in *S*-nitrosylation of both subunits of RuBisCO, which in turn inhibited the activity of this enzyme. Nitrosoglutathione (GSNO) at a concentration of 25 mM inhibited the enzyme activity in 20 % while 500 mM GSNO in 60 % (Abat et al. 2008). Process of *S*-nitrosylation is reversible and light sensitive. It can be assumed that 3 days after embryo treatment with NO both subunits of RuBisCO could be denitrosylated resulting in formation of fully active enzyme. As it was mentioned previously, NO increased photosynthetic activity of apple seedlings. *S*-nitrosylation is believed to prevent carbonylation, in contrast regarded as irreversible modification (Dalle-Donne et al. 2003). So, from this point of view, NO could act as a protective factor against protein oxidation. Moreover, RuBisCO synthesis correlates to N influx into leaves as was presented for *Eucalyptus globulus* seedlings (Suzuki et al. 2010). Extra N influx into leaves resulted in higher RuBisCO synthesis, thus NO could act not only as signaling molecule or protein modulator but also as non-direct stimulator of RuBisCO synthesis.

Sugars are known to take part in control of growth and development during the entire life cycle of plants. Signaling by carbohydrates includes action of sugars and sugar-derived metabolic signals (Rolland et al. 2002; Smeekens et al. 2010). During seed germination and seedling growth sugars modify nutrient mobilization, hypocotyl elongation, greening and expansion of the cotyledons (Rolland et al. 2002). Moreover, it is known as a link of Glc to ABA and ethylene-signaling pathways (Karve et al. 2012). High Glc concentration blocks switch from seed germination to seedling development (Cheng et al. 2002). On the other hand, transfer of young Arabidopsis seedlings germinating in the absence of Glc to Glc-containing media showed a stimulatory effect on root and shoot growth (Yuan and Wysocka-Diller 2006). It is accepted that Glc function is hormone-like and associated with hexokinase activity, which acts as its sensor. We observed fluctuations in concentration of soluble reducing hexose (detected as Glc units) NO treatment slightly increased content of reducing sugars in both cotyledons. These findings are in agreement to ones described for apple embryos treated with HCN, which stimulated glycolysis and increased Glc level during embryo germination (Bogatek et al. 1999). It is possible that NO also interact via Glc in establishment of autotrophy.

The electron microscopy studies of the upper cotyledons isolated from control seedlings and seedlings developed from NO-treated embryos (NO) or NO-treated control seedlings after 5 days of culture (5d+NO) indicated modifications in their ultrastructure. NO influenced chloroplast development, independently of the stage of ontogeny (embryos after isolation from the seed coats or abnormal seedlings). In NO-treated cotyledons chloroplasts were characterized by well-developed lamellar system. In the control, upper cotyledon (remaining white till the termination of the experiment) cells were small with proplastids rather than fully developed chloroplasts. It corresponds to previously described data indicating that dormancy alleviation initiated by cold stratification (and HCN release) led to cytological modification, described mostly for embryonic axis. Among them, accumulation of starch granules in cotyledons was the most frequently observed (Dawidowicz-Grzegorzewska 1989; Lewak 2011).

ROS, such as singlet oxygen (^1^O_2_) are involved in retrograde signaling during late embryogenesis of Arabidopsis seeds. These molecules play important role in plastid differentiation after seed germination. The effect of ^1^O_2_-mediated retrograde signaling depends on ABA, which is a positive regulator of plastid formation (Kim et al. 2009). NO stimulation of ROS accumulation in apple embryos was discussed above. ABA impact on chlorophyll synthesis was analyzed almost 30 years ago by Le Page-Degivry et al. (1987). Inclusion of ABA into the growing medium of isolated cotyledons resulted in enhanced chlorophyll biosynthesis and accelerated plastid development. We do not have data indicating influence of NO on ABA synthesis in apple cotyledons of growing seedlings. We can suspect that an increased ABA content could be associated with the progress of seedlings autotrophy. This conclusion comes from data by Bogatek et al. (2003) indicating ABA enlargement in apple embryos shortly treated with HCN.

To summarize, short-term (signaling) NO treatment stimulates autotrophy progress in young apple seedlings, independently of the time points of its application. This molecule stimulates chloroplast biogenesis, chlorophyll biosynthesis and in a consequence, photosynthetic activity. Mode of action of NO is linked to enhanced ROS and Glc level.

### *Author contribution statement*

AG and UK conceived and designed research. RB helped in research design. UK and KO organized and conducted experiments. KD ran part of experiments. UK and AG analyzed data and wrote the manuscript. All authors read and approved the final version of the manuscript.

## Abbreviations

cPTIO: 2-(4-Carboxyphenyl)-4,4,5,5-tetramethylimidazoline-1-oxyl-3-oxide
ROS: Reactive oxygen species
RuBisCO: Ribulose-1,5-bisphosphate carboxylase/oxygenase
SNAP: *S*-Nitroso-*N*-acetylpenicillamine
SNP: Sodium nitroprusside
